# Letter from J. B. Fares, M. D., Romeo, Mich.

**Published:** 1867-12

**Authors:** J. B. Fares

**Affiliations:** Romeo, Mich.


ROMEO, MICH., *5th December, 1867.*

PROF. J. A. ALLEN, M.D., Chicago:

*Dear Doctor:*—I noticed, in a recent No. of your JOURNAL, that you regard the indiscriminate use of caustics, in ulceration of the os uteri, as very reprehensible. I have, for a long time, regarded them as potent remedies, if judiciously applied, yet liable to be very improperly used. I have succeeded admirably in the treatment of the above mentioned disease, by pursuing a milder course than that usually adopted, and have concluded to give a sketch of the plan adopted, allowing you to judge whether it is worthy of insertion in your columns.

In chronic ulceration of the os uteri, I used the argent. nit. fus., at first, repeating in the usual interval of about five days, but very rarely find it necessary to make more than the second application. The subsequent treatment consists in the use of the following:—℞. argent. nit. crys. ʒss, aquæ dist., f.℥; M. fiat sol. Applied in the following manner:—First dry the secretions about the diseased surface (which is brought to view by means of the speculum) by the use of a fine sponge, moistened, and secured to a caustic-holder or probang; then with a camel's hair pencil cut short, and wound with a thread from the holder to within an inch of the point, in order to lessen its mobility; the above mentioned solution is carefully painted all over the diseased surface, until the characteristic whiteness is observed, repeating every third day; and after each application, in case there is evidence of the ulceration extending far within the cervix (which is found to be the case almost invariably in chronic cases), I inject about a-half fluid ounce of solution of the argent. of the following strength—viz.: five grains to the ounce. At the same time taking the precaution not to do this nearer than two or three days either preceding or following the menses.

In recent cases the lunar caustic is not used at all, the latter measures being fully adequate to cure.

I have found the above method much more satisfactory, both in point of time, and in lessening the liability to recurrence, than harsher measures such as are usually recommended.

Your obedt. Servant,

J. B FARES.

